# Supplementary material for: Are carbon emissions trading and green financial instruments synergistic? -Comprehensive quantitative research based on content analysis
Source: PLoS One. 2024 Mar 7;19(3):e0298601. doi: 10.1371/journal.pone.0298601 (PMC10919601; doi:10.1371/journal.pone.0298601)
Supplement: S1 Data — (ZIP) [file pone.0298601.s001.zip › Data/地级市Z_F工作报告.zip!/╕╩╦α/╛╞╚¬╩╨/└┤╫╘╣1⁄2╓┌║┼í╛┬φ┐╦╩2╛▌═°í┐_2012.docx]

各位代表：

　　现在，我代表市人民政府向大会作工作报告，请予审议，并请各位政协委员和列席人员提出意见。

　　2011年的工作及本届政府工作回顾

　　2011年，是国内外发展环境复杂多变的一年，面对通胀压力加大、宏观政策趋紧的形势，我们在市委的正确领导下，以科学发展观为统揽，深入贯彻党的十七大和十七届五中、六中全会精神，认真落实省委、省政府区域发展战略和市委的决策部署，攻坚克难，扎实工作，较好地完成了市二届人大七次会议确定的任务目标，实现了“十二五”规划的良好开局。预计全市完成生产总值480亿元，增长16%；全社会固定资产投资570亿元，增长30%；财政收入60亿元，增长18.8%，财政支出80亿元，增长21.8%；社会消费品零售总额105亿元，增长20%；城镇居民人均可支配收入17068元，增长13%，当地农民人均纯收入8030元，增长11%，移民人均纯收入2206元，增长20%；城镇登记失业率控制在3.19%，人口自然增长率控制在5.5‰以内；节能减排完成约束性指标；居民消费价格涨幅控制在6%以内。政府承诺的创业促就业、保障性住房建设等10件惠民实事有9件得到落实。今年工作的主要特点是：

　　1、项目建设成效显著。坚持把项目建设作为转方式、调结构、保增长的重要抓手，紧紧围绕“十二五”规划，加紧策划、对接、申报重大项目，固定资产投资持续稳定增长。全市开工建设各类项目1105项，其中：投资亿元以上项目104项，5000万元以上项目253项。加快交通基础设施建设，敦当二级公路建成通车，敦煌客运枢纽站投入使用，兰新铁路第二双线酒泉段、瓜星高速公路、酒嘉城际公路完成年度建设任务。水利工程建设取得重大突破，敦煌水资源合理利用和生态保护综合规划获国务院批复，北大河生态环境综合治理项目完成设计方案招标，灌区续建配套、病险水库加固、中小河流治理顺利推进。积极引进优势产业项目，成功举办承接东中部产业转移项目推介会，与24家中央企业签订战略合作协议，组团参加兰洽会、广交会等招商节会，签约合作项目189个，引进到位资金143.5亿元，增长19%。

　　2、工业实力不断增强。全面实施推进工业跨越式发展两个意见和六个方案，着力培育战略性新兴产业，做大做强传统优势产业，预计工业增加值达到210亿元，增长21%。扎实推进新能源基地建设，风电二期800万千瓦首批300万千瓦项目启动建设，65万千瓦大型国产化风机示范项目建成，43兆瓦光电项目并网发电，全省首个风光互补示范项目在玉门即将建成，新增光电装机200兆瓦，累计达到243兆瓦。840万千瓦火电项目列入国家电网规划，酒泉热电厂2×33万千瓦火电项目建成投运。100万千瓦抽水蓄能电站前期工作进展顺利，新增水电装机6.4万千瓦，累计装机达到32.9万千瓦。加大电网建设争取力度，提高电力输出保障能力，柳园330千伏变电站建成运行，敦煌-鱼卡-格尔木750千伏输变电工程、±800千伏直流输变电工程完成换流站选址和线路勘查。积极发展装备制造业，光伏产品制造实现零的突破，风机设备年生产能力达到300万千瓦。加快矿产资源勘查开发，启动第二轮矿产资源整合工作，一批现代高载能产业项目开工建设，矿产品采选冶炼占工业增加值的比重达到28.6%。合理布局建设高新技术产业园、循环经济产业园和资源综合利用产业园，“一区多园”的工业园区格局初步形成，酒泉工业园区申报国家级经济开发区通过商务部审核，报国务院待批。

　　3、现代农业加快推进。认真落实强农惠农政策，各级财政投入“三农”资金14.86亿元，增长12.2%。调整优化农业结构，新增蔬菜、制种、特色林果等高效作物面积23万亩。实施百万亩高效蔬菜发展规划，新建日光温室4700亩、塑料大棚2.2万亩，蔬菜面积达到50万亩，较上年增加12万亩。推进牛羊产业大县建设，新建养殖小区150个，调引良种羊4.4万只，肉羊饲养量达到590万只，规模养殖户达到2万户。提升农业产业化水平，新续扩建农产品加工项目24个，市级以上龙头企业达到77家，新增农民专业合作社45个，我市被确定为国家首批农业产业化示范基地。推进农村土地整治和农业集约规模经营，新增土地流转面积6.5万亩，累计达到29.7万亩，占承包面积的15.3%。开展农民工技能培训3.1万人，输转农村劳动力16.3万人，实现劳务收入14.8亿元。扎实推进新农村建设，100%的建制镇、95%的乡和82%的村完成规划编制，新建百户以上楼居式中心村32个，创建村容村貌示范点227个。

　　4、城市功能日趋完善。以提升城市品位为目标，坚持高起点规划、高标准建设、高水平管理，酒嘉一体化城市总体规划纲要通过评审，酒泉城市总体规划及部分区域控制性详规加紧编制。加大酒泉老城区改造力度，推进鼓楼东北片、金泉路东片综合开发。完善城市基础设施，新建集中供热管网5.3公里，铺设天然气管道28公里，完成供水分户改造3200户、供热计量改造31.8万平方米。积极创建园林城市，新增绿化面积52万平方米，城区绿化覆盖率达到34.9%。县市城区建设步伐加快，敦煌总干渠和瓜州九支渠水景改造、肃北引水入城工程、金塔东环路和阿克塞民主路改造、玉门石油大道和集中供热工程等重点项目建成。提高城市管理水平，加强市政设施、环卫绿化、供水供热和物业管理，开展城中村、城乡结合部环境综合整治，市容市貌进一步改善。

　　5、第三产业繁荣活跃。编制“十二五”服务业规划和通道经济发展规划，推进国家服务业综合改革试点工作，落实引导资金、考核办法和扶持政策，推动服务业快速发展，第三产业增加值达到160亿元，增长14%。市场规模进一步扩大，春光市场二期扩建、玉门再生资源市场等项目建成，兑付家电补贴1121万元，销售家电下乡产品3.9万件，家电以旧换新9000余件，实现销售收入1.5亿元。物流、研发、设计等生产性服务业快速发展，国家风电设备质量监督检验中心（实验室）落户酒泉，3家风光电技术研究机构成立。推进金融体系建设，与招商银行签订金融合作协议，交通银行在酒泉设立分支机构，新增9家小额贷款公司和8家融资担保机构。大力扶持旅游业发展，敦煌旅游股份公司挂牌成立，成功举办敦煌行·丝绸之路国际旅游节闭幕式，积极开展景区景点、宾馆饭店晋级达标活动，旅游服务质量稳步提高。全市接待游客511.6万人次，增长39%；实现旅游收入43.4亿元，增长41%。民营经济蓬勃发展，全市非公企业达到4340户，个体工商户达到3.6万户，非公经济增加值占全市经济总量的43%，提供了31%的非农就业岗位，对地方财政的贡献达到23.7%。

　　6、生态建设取得实效。坚持把节水作为生态建设的关键，制定加快水利改革发展意见，全面推行差别水价制度和严格的水资源管理制度，新增节水灌溉面积10万亩，累计达到158万亩。集体林权制度改革全面完成，181万亩集体林木确权到户。继续实施封滩育林、退耕还林、退牧还草等生态建设工程，846万亩生态公益林得到有效保护。认真落实淘汰落后产能政策和甘肃省循环经济总体规划，强制推行差别电价政策，对13户重点耗能企业实行预警监测，依法关停小冶炼企业12户，淘汰钢铁落后产能25万吨，单位GDP能耗下降4%。编制“十二五”环境保护规划和主要污染物减排规划，污染源自动监控中心、医疗废物集中处置中心投入使用，酒泉、玉门、敦煌污水处理厂和玉门炼化总厂污水处理工程达标运行，瓜州、肃北、阿克塞垃圾处理项目基本建成，化学需氧量等四项减排指标全面完成。落实农村环保以奖促治政策，51个规模化畜禽养殖场列入“十二五”减排项目，14个村镇确定为全国生态创建示范点，48个村实施村容村貌联片整治项目，农村生态环境得到改善。

　　7、社会事业协调发展。科技创新八项行动深入推进，实施科技项目136项，现代制种及装备技术示范等项目列入国家星火计划，2.5兆瓦直驱永磁风机研发等项目列入省科技专项，申请各类专利296件，全国科技进步先进市创建通过国家验收。启动实施教育体制改革和学前教育三年行动计划，推进农村幼儿园标准化建设，加快实施校安工程，39个重点项目完成主体工程，改造危旧校舍3万平方米，建成市级标准化学校20所，选派300名城乡优秀教师进行双向交流，“两基”工作通过国家检查验收。深化医药卫生体制改革，基本药物和医用耗材“零差率”扩大到县级公立医院和妇幼、疾控、计生等卫生服务机构。医疗卫生条件继续改善，市医院门诊综合楼、市二院住院部大楼、市中医院制剂楼等25个新续扩建项目建成使用。投入公共卫生服务均等化资金2019万元，人均补助标准达到25元，农村医疗救援、健康档案等10项公共卫生服务实现全覆盖。开展“婚育新风进万家”、“生殖健康服务进家庭”、“利益导向示范区建设”活动，人口和计划生育工作水平提高。文化事业健康发展，以庆祝建党90周年为主题，广泛开展群众文化活动，肃州区银达镇文化工作经验在全国推广，酒泉文化中心完成方案设计，文化馆、图书馆和乡镇文化站免费向公众开放，玉门文化“三馆”建成。启动实施全民健身五年计划，体育惠民工程扎实推进，成功举办第三届中小学生田径运动会，承办敦煌全国沙滩排球巡回赛、第六届玄奘之路戈壁挑战赛等体育赛事。数字电视转换和20户以上自然村广播电视“村村通”工程全面完成。深入开展和谐社区创建活动，建成国家级、省级示范社区10个，农村社区建设试点村达到55个。强化安全生产监管，推行安全隐患挂牌督办、指标预警机制，深入开展安全生产事故遏制行动、重点行业专项整治和拉网检查，安全生产四项控制指标同比下降。“六五”普法启动实施，“三调联动”机制不断完善。开展矛盾纠纷排查和“两访”治理活动，1140件矛盾纠纷、27件信访积案得到调处化解。加强社会治安综合治理，推进“天眼”、“百卡”工程建设，城乡警务条件明显改善，玉门公安大楼主体工程完工。强化食品、药品质量监管，重点整治滥用食品添加剂和无证生产等非法行为，保障群众健康安全。全国双拥模范城创建通过国家验收，6个县市被命名为省级双拥模范城（县）。继续开展民族团结进步宣传月项目帮扶活动，落实帮扶资金3123万元，肃北县、阿克塞县分别被确定为全国“兴边富民”行动重点县和全省共同团结奋斗、共同繁荣发展示范县。第二次经济普查和第六次人口普查工作完成，统计监测服务水平进一步提高。防震减灾和人防、边防工作深入推进，妇女、儿童、台侨、宗教、档案等工作取得新的成绩。

　　8、社保水平不断提高。加大就业扶持力度，落实就业补贴资金1.1亿元，选拨1089名高校毕业生到基层就业，新增城镇就业1.4万人。深入推进创业促就业行动，新增小额贷款担保基金3030万元，为3.6万名创业人员和33户企业发放贷款13.3亿元，带动城乡就业6.1万人。扩大城镇职工基本养老、医疗、失业、工伤保险覆盖面，启动实施城镇职工生育保险制度，推行城镇户籍临时工、原“五七工”、“家属工”养老保险制度，原国有企业"老工伤"人员全部纳入工伤保险统筹，玉门市列入国家首批城乡居民养老保险试点，金塔、肃北、阿克塞新农保参保率均达到95%以上，被征地农民和村干部养老保险实现全覆盖。提高城乡居民医疗保险补助标准和住院报销比例，全面落实新农合门诊统筹制度，新农合参合率和城镇居民医保覆盖面均达到98%，社区卫生服务机构运行经费和人员工资得到保障。城乡低保标准分别提高10%和22%，为16.9万名城乡低保对象发放最低生活保障金1.7亿元，为“三老一少”发放生活补贴511万元。五保供养标准提高到2400元，保障人数2992人，集中供养率达到20.4%。完善城乡居民临时救助和医疗救助制度，为困难家庭、优抚对象、建国前老党员发放临时救助金和生活补贴2300万元，为1.9万名城乡居民发放医疗救助金1558万元。加快发展社会福利事业，市儿童福利院、肃州和敦煌社会福利院完成标准化创建，酒泉未成年人救助站、金塔敬老院等项目建成，全市65%的社区建立居家养老服务站。保障性住房建设超额完成年度任务，新建廉租房1020套、棚户区改造安置房1080套，建设公租房和“两限”房1026套，为1.08万户困难家庭发放了住房租赁补贴，对近年建成的7126套廉租房、4937套经济适用房进行公开分配，新建商品房价格涨幅控制在2.33%，低于全省平均水平。加强蔬菜、肉类、禽蛋价格监控，落实蔬菜发展基金600万元，设立惠民肉菜店10个。改善农村居民生产生活条件，新建和改建农村公路1000公里，改造困难群众和残疾人危房20450户，安全饮水工程又使5万农村居民受益。

　　9、移民帮扶力度加大。深入实施移民区发展两个五年规划，加大扶贫开发力度，争取扶持资金1.7亿元，增长18.4%。加快移民乡村基础设施建设，营造农田防护林7644亩，改良盐碱地2.3万亩，衬砌渠道253公里，铺筑道路197公里，改造移民住宅5310户，移民乡村电话普及率达到97%，有线电视覆盖率达到98%。因地制宜发展特色产业，新建日光温室133座，种植棉花、药材、瓜果等经济作物17.7万亩，新建暖棚圈舍1504座，调引基础母羊1.6万只，牛、羊存栏量分别达到1797头和15.3万只，增长73%和12%。引导鼓励移民外出务工，开展移民技能培训3.2万人，输转劳动力2.1万人次。完善移民社会保障制度，落实医疗救助、救灾救济等倾斜政策，8.2万贫困移民纳入农村低保范围，新农合参保率达到98%。

　　10、行政效能进一步提升。加快转变政府职能，全面完成市、县、乡机构改革。深入开展创先争优活动，政府工作效能和服务水平继续提高。落实重大决策公开听证和责任追究制度，健全公众参与、专家论证、风险评估、合法性审查决策机制，聘任行政执法社会监督员30名，政务公开面达到100%。发挥“市长信箱”作用，广泛了解社情民意，群众反映的一些问题得到重视和解决。规范政务大厅运行管理，整合组建资金结算、人才交流、住房资金、房产交易、车辆管理、社会保险六个分中心，入驻部门和单位40个，受理办件33万件，办结率达到99.9%。坚持依法行政，强化依法行政责任考核，与49个政府部门和驻酒单位签订依法行政责任书。自觉接受人大法律监督、工作监督和政协民主监督，办理人大代表意见建议和政协提案306件，办结答复率达100%。加强政府廉政建设，推行政府投资项目集中代建制、资金监督管理和规范市场中介组织从业行为三项制度。开展公务卡结算试点改革，严格控制“三公”经费，市级预算管理单位国库集中支付和会计集中核算实现全覆盖。加强行政监察，严格落实工程招投标、土地矿产出让、产权交易和政府采购制度，继续开展“小金库”专项治理，确保权力在阳光下运行。加大审计监督力度，对预算执行和其他财政收支情况及34名领导干部进行了审计，对违纪违规问题进行了处理和整改。各位代表，本届政府任期已满。五年来，我们积极应对金融危机挑战，努力克服各种自然灾害影响，把握宏观经济走势，抢抓国家政策机遇，坚持“两抓整推”总体思路，团结带领广大干部群众奋力拼搏、艰苦创业，超额完成了“十一五”规划和市二届人大一次会议确定的目标任务，促进了酒泉经济社会跨越式发展。

　　--过去的五年，我们以科学发展为统揽，着力培育优势产业，综合经济实力明显提升。全市生产总值年均增长15.1%，人均GDP突破6000美元，财政收入年均增长33.7%，经济总量跃居全省第二。实施振兴工业“6+2”行动计划，工业规模迅速扩大，工业占GDP的比重由32.9%提高到41.7%，对经济增长的贡献达到52%。大力发展“一特四化”现代农业，农业结构进一步优化，高效特色产业面积达到183万亩，占耕地面积的73.2%，肉羊饲养量居全省第一。服务业繁荣活跃，区域性商贸中心、国际旅游城市的地位初步形成，旅游收入占GDP的比重提高到9%，我市被国家列为甘肃省唯一的服务业综合改革试点城市。

　　--过去的五年，我们以优化环境为保障，狠抓基础设施和生态文明建设，可持续发展能力明显增强。一批交通、水利、生态等事关全市长远发展的重大项目相继建成，累计完成固定资产投资1581亿元，年均增长43.8%。交通设施显著改善，嘉峪关-瓜州高速公路、瓜州-敦煌二级公路建成通车，改造新建农村公路6465公里，建成和在建高速公路488公里。城乡面貌明显改观，城市建成区面积达到90平方公里，新增20平方公里，城镇人均住房面积达到30.8平方米，增加9.1平方米，农村砖木、砖混结构住房比重达到41.8%，提高8.8个百分点，城镇化率由46.4%提高到50.1%。生态环境保护取得实效，累计关井412眼，压田4.5万亩，新增节水能力5000万立方米；完成人工造林49.3万亩，封滩育林110万亩，森林覆盖率由1.87%提高到3%。万元GDP能耗和主要污染物排放控制在约束性指标以内，公众对城市环境保护满意率达到86.4%。

　　--过去的五年，我们以构建和谐酒泉为基点，协调发展各项事业，促进了社会全面进步。全市用于科教文卫等社会事业支出年均增长26.8%，占财政支出的比重由25.9%提高到31.3%。科技创新步伐加快，实施科技项目980个，科技进步对经济增长的贡献率达到50%。按照“教育资源相对集中”的思路，优化教育布局结构，完成55个乡镇的中小学布局调整任务，撤并农村中小学288所，初步形成“高中集中在城区、初中集中在城镇、乡镇集中办好九年一贯制学校和完全小学”的办学格局，义务教育“四率”保持在部颁标准以上，高中阶段入学率稳定在93%以上。坚持“医疗资源切实下沉”的思路，实行医疗资源向大病倾斜、向困难群众倾斜、向城乡非职工倾斜，基本实现初级卫生保健目标，全市乡镇、村组、社区卫生服务机构和县级公立医院实现基本药物“零差率”，基层医疗机构药品费用下降45.6%，新农合覆盖率提高到98.2%，我市被评为全国人口与计划生育综合改革示范市。大力推进文化创新，满足群众文化需求，文化馆（室）和“农家书屋”建设成效显著，县乡村文化信息共享工程实现全覆盖。广播电视综合覆盖率达到98%，有线电视和数字电视入户率分别达到77%和52%。

　　--过去的五年，我们以改善民生为根本，认真落实惠民政策，群众生活水平明显提高。城镇居民人均可支配收入年均增长13.7%，农民人均纯收入年均增长13.9%，移民收入年均增长20.4%，均高于全国、全省平均水平，城乡居民家庭财产普遍增加，人民生活更加殷实。全市城乡居民收入差距缩小到2.1:1，低于全国、全省平均水平，城乡一体化总指数达到7.8，居全省第二。社会保障制度基本建立，最低生活保障制度和医疗保险制度实现全覆盖，农村养老保险试点稳步推进，城乡社会救助体系不断健全，实行了“三老一少”、低保边缘户生活补贴制度和寄宿生交通费、伙食费“双补”制度，提高城镇职工、城乡居民基本医疗保险待遇水平，现任村干部工资、离任村（社区）干部生活补贴和村委会办公经费得到保障。全市参加城乡养老、医疗、失业、工伤等各类保险人数达121.5万人次，社会保险综合覆盖面达到85%。市政府承诺的48件惠民实事得到较好落实，新建各类保障性住房14385套，发放租赁补贴11434户，改造农村危旧房、残疾人住房3.7万户，解决了32万农村居民的安全饮水问题，建成沼气池3.8万座，全市25%的农户用上了洁净能源。

　　各位代表，五年的历程很不平凡，五年的成绩来之不易。这是省委、省政府和市委坚强领导的结果，是市人大、市政协和社会各界大力支持的结果，是全市人民辛勤努力、共同奋斗的结果。在此，我代表市政府，向各位人大代表、政协委员和全市广大干部群众，向各民主党派、工商联、人民团体、离退休老同志，向驻酒人民解放军、武警官兵和公安干警，向中央、省属驻酒单位以及所有关心、支持酒泉发展的各界人士表示衷心的感谢，并致以崇高的敬意！

　　在总结成绩的同时，我们也清醒地认识到，政府工作与群众期待还有一定差距，当前还存在一些不容忽视的问题，主要表现在：风电二期项目建设进度较慢，装备制造业发展受到一定影响；肉菜蛋等生活必需品价格涨幅较大，城乡居民特别是低收入家庭的生活负担加重；政府承诺的十件实事之一的城区集中供热和天然气入户工程没有完成年度建设任务。这些问题，既有宏观政策趋紧、项目审批放缓、融资难度加大等客观原因，也有我们主观努力不够的因素，尤其是对经济走势的预见性不强，一些工作创新不够、措施不力、效率不高，影响了重点工作的推进。对此，我们将高度重视并加以解决。

　　今后五年的目标任务

　　今后五年，是全面建设小康社会的关键时期，也是转变经济发展方式、实现酒泉跨越式发展的攻坚时期。国家实施新一轮西部大开发战略，出台支持甘肃发展的47条意见，这有利于我们争取国家政策投资，加快基础设施、生态环境建设和社会事业发展；国家大力扶持清洁能源发展，将我市确定为千万千瓦级风电基地、百万千瓦级光电基地和风光电装备制造产业化基地，这有利于我们做大做强新能源及装备制造业；国家将我市列为服务业综合改革试点城市，并赋予新疆加快发展的优惠政策，这有利于我市发挥承东接西、展南拓北的区位优势，加快通道经济和服务业发展；省委、省政府区域发展战略将我市确定为“两翼齐飞”的重要一翼，这有利于我们发挥比较优势，实现率先发展。

　　今后五年政府工作的总体要求是：以科学发展观为统领，深入贯彻省委、省政府区域发展战略和市三次党代会精神，以建设富裕、文明、和谐、幸福酒泉为目标，以转变发展方式为主线，以改革开放和科技创新为动力，以重大项目为支撑，以新能源及新能源装备制造业为牵引，以保障和改善民生为己任，做精第一产业，做强第二产业，做大第三产业，统筹推进经济建设、政治建设、文化建设、社会建设和生态建设，打造“西翼”增长极，实现发展新跨越，为全面建设小康社会打下坚实的基础。

　　主要奋斗目标：通过五年的努力，实现“五个翻番”和“五个提升”，全市生产总值、工业增加值、财政收入、固定资产投资和社会消费品零售总额五项指标在“十一五”基础上翻一番，财政收入“过百亿”，生产总值“超千亿”；城乡居民收入、社会文明程度、社会管理水平、公共服务水平和人民群众幸福指数明显提升，城镇居民可支配收入和农民人均纯收入年均保持两位数增长，移民人均纯收入达到全省农民人均收入水平，城镇化率每年提高1个百分点，节能减排完成约束性指标，城镇登记失业率控制在4%以内，人口自然增长率控制在5.5‰以内。

　　为实现上述目标，我们必须顺应人民群众过上美好生活的新期待，准确把握不断变化的新形势，抢抓加快发展的新机遇，树立求真务实、开拓进取的新风尚，推动全市经济社会实现跨越式发展。

　　--建设“富裕酒泉”。按照“基础先行、产业支撑、强市富民”的思路，努力实现经济总量明显扩大、地方财力明显增强、城乡居民收入明显增加的目标。坚持工业化与城镇化相促进、三次产业相协调、发展成果与民众福祉相统一，加快转变发展方式，提高经济运行质量，着力构建新型工业、现代农业和服务业发展新格局。大力发展新能源及配套产业，推进工业转型升级，力争把酒泉建成全国重要的新能源示范基地、装备制造基地、资源综合利用基地和新型工业化示范基地，全市风电、光电、火电装机和新能源装备制造业销售收入完成“十二五”规划目标，工业占GDP的比重提高到48％以上。加快构建现代农业新体系，打造特色农产品生产加工基地，高效产业面积达到80%以上，农产品转化增值率达到65%以上，努力实现农业增效、农民增收和农村环境面貌显著改善的目标。以开展国家服务业综合改革试点为契机，改造提升传统服务业，加快发展现代服务业，形成服务业与工业并重发展格局，把酒泉建成全国重要的商贸物流中心，力争第三产业增加值占GDP比重提高到37%，吸纳就业占全社会从业人员的比重提高到42%以上。改善基础设施条件，构建“四横四纵”铁路网络，提升县乡村道路等级，形成完善、稳定、便捷的交通体系。推进城乡一体化、酒嘉经济一体化进程，着力建设“宜居、宜业、宜商、宜游”的戈壁明珠城市，为建设“富裕酒泉”营造良好环境。

　　--建设“文明酒泉”。以十七届六中全会精神为指导，高度重视文化软实力建设，坚持用先进文化引领发展方向，做到物质文明与政治文明、精神文明、生态文明协调发展。推进社会主义核心价值体系建设，加强社会公德、职业道德和家庭美德教育，提升城乡居民文明素质、公务人员服务水平和企业的社会责任意识。深入开展精神文明创建活动，建成全国文明城市和国家级园林城市。深化文化体制改革，推动文化事业大发展大繁荣，把文化产业培育成新的经济增长点。坚持“生态立市”战略，全面实施敦煌水资源合理利用与生态保护综合规划，建设国家级湿地补偿试点、生态补偿实验区、荒漠化治理示范区，构建“一区四屏”生态安全屏障。坚持依法、科学、民主决策，提高公正、文明执法水平，努力建设法治政府、诚信政府、阳光政府和廉洁政府。

　　--建设“和谐酒泉”。全面发展社会事业，加强和创新社会管理，努力实现收入差距逐步缩小、公共服务不断扩大、群众安全感明显增强的目标。调整收入分配关系，增加低收入家庭收入，扩大中等收入群体比重。建立多元化矛盾纠纷排查调处机制，畅通群众利益诉求渠道，认真解决群众关心的热点难点问题，从源头上预防和减少信访事件发生。深入实施“六五”普法规划，积极开展法律援助，保障弱势群体合法权益。严格落实安全生产责任制，开展重点领域专项整治，严防重特大事故发生。推进公安“三基工程”和“五大建设”，完善治安防控体系，严厉打击犯罪活动，确保实现“发案较少、秩序良好、社会稳定、群众满意”的目标。狠抓移民扶贫开发，改善移民生产生活条件，基本实现移民脱贫目标。

　　--建设“幸福酒泉”。坚持普惠、实惠、常惠相结合，提高民生保障水平，使城乡居民劳有所得、病有所医、老有所养、住有所居，人民群众幸福指数明显提升。实施更加积极的就业政策，以发展扩大就业、以政策促进就业、以创业带动就业、以培训提升就业，加大就业困难人员的帮扶力度，做好高校毕业生就业和农村劳务输转工作，促进劳动力就业本地化，城镇登记失业率控制在4%以内。按照“广覆盖、保基本、多层次、可持续”的要求，全面普及城乡社会养老保险制度，提高失业、工伤、生育保险和最低生活保障水平，推行医疗保险门诊统筹、异地就医结算、医疗保险关系转移接续等制度，构筑更加完善的社会保障体系。抓好“菜篮子”工程建设，扩大粮油储备规模，建立肉类储备制度，保障生活必需品的市场供应和价格稳定。强化房地产市场监管，加快保障性住房建设，新增各类保障性住房90万平方米。强化食品安全监管，规范医药市场秩序，加强饮食卫生源头治理，切实保障人民群众健康安全。

　　2012年工作建议

　　2012年，是贯彻落实市三次党代会精神的第一年，也是深入实施“十二五”规划的重要一年。做好明年的政府工作，对建设富裕、文明、和谐、幸福酒泉具有十分重要的意义。

　　全市经济社会发展的预期目标是：生产总值增长16%，固定资产投资增长30%，财政收入增长16%，社会消费品零售总额增长20%，城镇居民人均可支配收入增长13%，当地农民人均纯收入增长11%，移民人均纯收入增长20%，居民消费价格涨幅控制在省上确定的目标以内，城镇登记失业率控制在4%以内，人口自然增长率控制在5.5‰以内，万元生产总值能耗降低4%，主要污染物排放完成约束性指标。实现上述目标，应重点抓好九个方面的工作：

　　1、加快构建新型工业体系。坚持实施工业强市战略，发展壮大地方工业实力，力争工业增加值增长20%以上。抓好风电二期800万千瓦首批300万千瓦项目建设，新增风电装机200万千瓦，完成风电二期后续500万千瓦项目前期工作。加快建设以敦煌、金塔、肃州为重点的光电产业基地，推进风光互补、风光储一体化项目建设，新增光电装机150兆瓦。开工建设明沙窝调峰火电项目，完成常乐、红柳洼、酒泉热电厂二期和昌马电厂前期工作。加快柳沟峡80万千瓦、玉门昌马水库抽水蓄能电站前期工作，新增水电装机7万千瓦。着力破解电力输出瓶颈，开工建设敦煌-鱼卡-格尔木750千伏输变电工程，做好酒泉-株洲±800千伏特高压直流输变电工程前期工作。引大做强风光电装备制造企业，推进风电装备成套化、高端化和数字化，形成光伏组件生产能力100兆瓦，实现装备制造业销售收入200亿元。支持玉门油田争取炼化扩容和石油储备库项目，积极引进冶金、焦化、硅材料等现代高载能产业项目，理性承接东中部地区产业转移，实现能源就地消纳和资源循环利用。加快特色工业园区发展，抓好市管园区和县市工业集中区建设，大力推进“飞地建园”，增强聚集带动效应。认真落实中小企业扶持政策，培育壮大产业集群，新增销售收入亿元以上工业企业8户、千万元以上工业企业25户。

　　2、大力发展现代农业。以服务城市、富裕农民为目标，加快发展节水高效特色农业，促进农业转型和农民增收。加大惠农支农力度，完善以奖代补措施，落实草原生态奖补政策，财政支农资金增长10%以上。调整优化种植结构，新增现代制种、特色林果等高效产业面积10万亩，新增3000元以上高效田5万亩。抓好“菜篮子”基地建设，新建日光温室4000亩，新增大棚蔬菜1.6万亩。提高牛羊大县建设水平，新建规模养殖小区50个，新增肉牛1万头、肉羊30万只。实施30万亩甘草基地和精深加工项目，新增中药材种植面积10万亩。扶持壮大龙头企业，新增销售收入5000万元以上龙头企业2户，农产品加工增值率达到53%以上。创新农民专业合作社，构建集融资、信息、产供销为一体的新型专业合作社。开展农业科技培训20万人次，完成农民技能培训2万人，输转农村劳动力16万人次。积极推进标准化生产，完善农产品质量安全监督检测体系和畜禽疫病防控体系，农产品质量安全合格率达到99%以上。加快土地经营权流转，新增土地流转面积3万亩。扎实推进新农村建设，抓好“一池三改”、村组道路硬化和农村危旧房改造，整治和提升村容村貌示范点200个、示范区10个，建设百户以上社区化中心村30个。深入实施移民扶贫开发规划，争取落实各类扶贫资金1.5亿元，完成10个村的整村推进建设任务，整治改良土地3万亩，发展特色种植5万亩，促进移民收入持续增长。

　　3、狠抓重大项目建设。坚持发展抓项目不动摇，强化项目包抓包挂责任制，完善协调推进、督查督办和考核奖惩机制，着力引进培植税源、吸纳就业的产业项目，抓好基础设施、生态建设、社会事业、民生工程等重点项目，增强投资对经济增长的拉动作用。在生态水利建设上，全面推进敦煌水资源合理利用与生态保护项目，完成“引哈济党”前期工作，争取开工建设，启动北大河生态综合治理工程。继续实施农田水利重点县建设、中小河流治理、水库和水闸除险加固等工程，抓好党河、洪临等灌区续建配套与节水改造。在交通设施建设上，加快兰新铁路第二双线、肃阿二级公路、桥红二级公路建设进度，酒嘉城际一级公路和瓜星高速公路建成通车，酒泉客运枢纽站建成使用，加快敦格铁路、酒航高速、瓜州-当金山高速等重大项目前期工作。加强对外交流合作，提高招商引资质量，认真筹备参加“兰洽会”等招商节会，力争引进到位资金200亿元以上。

　　4、加快城镇建设步伐。以建设国际明星城市和戈壁明珠城市为目标，健全完善城市规划体系，修编完善酒泉市城市总体规划，编制完成新城区二期控制性详规，科学布局学校、医院、市场等公共服务设施，完成酒泉市区及金塔、瓜州、肃北、阿克塞城市总体规划报批工作，城乡规划实现全覆盖。加快酒泉老城区改造和新城区二期配套建设，抓好新建水源地和第二、第三污水处理厂建设，实施世纪广场周边及世纪大道景观带亮化工程。编制酒泉城区水系景观规划，营造水网环环相通、水体处处流动的城市景观，改善城市人居环境。实施中心城区增绿工程，新建城市各类绿地114万平方米，敦煌创建为国家级园林城市，瓜州、肃北创建为省级园林城市。推行城市精细化管理，加快数字城市建设，实现城市管理全方位覆盖和全时段监管。加强市容市貌、违章建筑、城市交通和公共秩序专项治理，深入实施“城中村”、城市出入口、城乡结合部环境综合整治，继续开展全国文明城市创建活动。创新城乡一体化发展机制，促进基础设施向农村延伸、公共服务向农村覆盖、农村人口向城镇转移，新增城市人口1万人，城镇化率提高1个百分点。

　　5、推进服务业和通道经济发展。把大力发展服务业作为优化产业结构的突破口，抓好服务业综合改革试点工作，优先发展现代服务业，壮大提升传统服务业，力争第三产业增加值增长14%。加快中心商贸区、商业聚集区和便民商业服务点建设，继续实施“万村千乡”市场工程，拓展社区、乡村商业网点，拉动消费持续增长。促进通道优势和产业优势融合互动，积极发展通道经济，引大做强物流企业，加快建设酒嘉现代物流园，推进物流业集团化发展、连锁化经营、网络化管理。做好“引行入酒”工作，深化农村金融机构改革，培育壮大村镇银行，扶持发展小额贷款公司和融资担保机构，加快敦煌旅游、巨龙供销、奥凯种机上市步伐。引导培育新兴服务业，抓好生产性服务业平台建设，完善科技、信息、商务等中介服务组织。加快旅游业发展，打响“敦煌飞天、酒泉航天、风光能源”三大品牌，加快数字莫高窟、瓜州锁阳城、阳关景区二期等项目建设进度，抓好玉门油田矿产遗址公园、风电长廊观光区、肃北梦柯冰川开发建设，创建一批星级饭店、星级农家园，争取将鸣沙山·月牙泉创建为5A级景区。举办敦煌行·丝绸之路国际旅游节和飞天文化旅游节，抓好旅游特色村镇建设，拓展国内外旅游市场，力争接待游客620万人次，增长20%，实现旅游收入52亿元，增长20%。

　　6、加强生态环境保护。深入实施生态立市战略，稳步推进水权、水价、用水制度改革，严格控制用水总量，提高用水效率，新增高效节水灌溉面积2万亩。继续实施封滩育林、禁牧休牧工程，抓好疏勒河、党河、黑河流域生态治理及重点水源地保护。开展全民植树活动，完成人工造林10万亩，义务植树320万株。加快移民区农田林网建设，促进移民区生态修复。落实节能减排目标责任，强化工程减排，推动结构减排，细化管理减排，推广资源节约、替代和循环利用技术，淘汰落后产能，提升污染治理水平。加快金塔、瓜州等县市污水处理项目建设，开工建设敦煌市污水处理厂提标改造工程和医疗废物处置中心项目，确保完成节能减排任务目标。开展环境优美村镇创建活动，抓好规模化畜禽养殖场环境整治。

　　7、统筹发展社会事业。以人的全面发展和社会公平正义为目标，完善公共服务体系，促进公共服务均等化。坚持教育优先发展，全面落实教育中长期改革和发展规划纲要，加快校安工程建设，改善移民乡、农垦团场办学条件，优化城区学校布局，努力缓解班额过大的问题。加强学校精细化管理和校园安全管理，新建市级标准化学校20所。巩固提高“两基”成果，大力发展学前教育，新续扩建农村幼儿园30所。实施国家示范性高职院校建设项目，拓展新能源技术学院办学领域。加快科技创新和成果转化，推进科技特派员基层创业工程，组织实施科技项目100项，示范推广新兴实用技术150项，建成市级以上工程技术研究中心15家。深化医药卫生体制改革，完善基本药物制度，全市县及县以下医疗机构全面实现基本药物“零差率”。大力发展中医药事业，继续实施医疗机构中医科、中药房标准化建设。健全基层医疗卫生机构运转保障机制，完善突发公共卫生事件应急处置预案。深化人口与计划生育综合改革，加强移民和流动人口计生管理，提高优质服务水平。开工建设酒泉文化中心，配套完善县级文化“三馆”和乡镇文化站设施设备，积极推进村组文化室建设。加强文化遗产保护，审定出版《酒泉通史》，启动酒泉历史文化丛书编写工作。举办第七届玄奘之路戈壁挑战赛，承办全国沙滩排球巡回赛和第五届青少年轮滑巡回赛。完成广播电视网络整合工作，实施肃北、阿克塞“西新”工程、农村电影放映工程和20户以下自然村广播电视“村村通”工程。启动建设气象服务"村村响"信息网络平台。认真落实少数民族和民族地区跨越发展的意见，大力开展民族团结进步创建活动，深入实施肃北县兴边富民“十二五”规划，抓好阿克塞“两个共同”示范县建设，促进民族团结与宗教和顺。开展双拥创建和军民共建活动，推进残疾人社会保障和服务体系建设，新建县级托养机构2个、阳光家园5个。发展慈善福利事业，实施儿童福利救助“蓝天计划”项目，做好统计、供销、妇女、儿童、台侨、档案、外事、人防、边防及防震减灾工作。

　　8、着力解决民生问题。坚持把充分就业作为经济社会发展的优先目标，高度重视高校毕业生就业工作，鼓励高校毕业生到农村、社区和企业就业，开展城乡困难人员就业援助，扩大创业促就业小额担保贷款规模，新增城镇就业1万人。健全城乡社会保障体系，落实城镇职工基本医疗、工伤保险、失业保险市级统筹制度，实施城乡社会养老保险试点，建立城镇居民基本医疗保险门诊统筹制度，完善新型农村合作医疗制度，巩固城乡低保提标扩面成果。推进社会福利院、农村敬老院建设，做好“三老一少”、五保户、低保边缘户救助工作，提高优抚对象补助标准。把平抑物价作为宏观调控的重点，适时启动价格补贴联动机制，建立和完善价格调节基金制度。加强房地产市场监管，加大保障性住房建设力度，努力解决困难群众和干部职工的住房问题。加强粮食、蔬菜、肉类等农产品市场流通和储备体系建设，推进农超对接、产地直销，保障农产品市场供应和质量安全。严格落实安全生产“一岗双责”制度，开展重点领域专项整治，严防重特大事故发生。高度重视食品、药品源头监控，保障人民群众身体健康。坚持用群众工作统揽信访维稳工作，畅通群众利益诉求渠道，健全重大工程项目和政策制定的社会稳定风险评估机制。深入开展普法教育和依法治理，抓好刑释解教人员安置帮教和社区矫正工作，推进法律服务向基层延伸、向非诉讼领域拓展。加强社会管理创新，扎实开展“打四黑、除四害”专项行动，严厉打击各类违法犯罪活动，保障群众生命财产安全和社会大局稳定。

　　切实解决民生问题，办好十件惠民实事：一是实施“千名大学生企业就业计划”，采取政府补贴基本生活费、企业缴纳社会保险和发放岗位工资的办法，选拔1000名高校毕业生到企业就业；二是加快实施校舍安全工程，改造危旧校舍10万平方米；三是继续实施农村安全饮水工程，再解决5万名农村居民安全饮水问题；四是新建、改造农村道路600公里；五是新建各类保障性住房3500套，棚户区改造5.5万平方米，发放租赁补贴1万户；六是改造农村困难群众危房1万户、残疾人危房500户；七是全面推行城乡居民社会养老保险制度，16岁以上城镇非从业居民和农村户籍人口全部纳入养老保险，60岁以上城镇非从业居民和农村户籍老人每月发放基础养老金60元，参保率达到85%以上；八是加快实施酒泉城区集中供热和天然气入户工程，实现市区联网供热，新增天然气用户1万户；九是在乡镇、街道设立视频监控点1450个，在重要路段及道口设置智能卡口26个；十是政府补贴资金，在城区新建30个便民肉菜销售点，规范现有蔬菜市场管理，减少中间环节，实现菜农与市民对接。

　　9、加强政府自身建设。深入开展创先争优活动，切实改进机关作风，提高行政服务效能。完善民主议事决策制度和重大决策专家论证制度，提高科学决策水平。自觉接受人大法律监督、工作监督和政协民主监督，高效办理人大代表意见建议和政协提案。全面推行依法行政责任制，进一步规范行政执法权，落实重大行政行为审查、备案制度。简化行政审批程序，规范行政许可收费，落实民间资本准入政策，完善非公经济扶持措施，减轻民营企业负担，为市场主体营造公平竞争的发展环境。扩大政务公开，完善信息披露和办事程序，增强政府工作的透明度。加强惩治和预防腐败体系建设，抓好重大项目、重点领域、重要环节的监管，强化行政监察和审计监督，增强公务员的服务意识、责任意识和廉洁意识，提高公务员队伍整体素质。

　　各位代表，面对新的目标、新的使命，我们深感任务艰巨、责任重大。我们有信心、有决心在市委的正确领导下，团结带领全市广大干部群众，开拓创新，奋力拼搏，全面完成市三届人大一次会议确定的任务目标。
